# Comparative Proteomic Analysis of Wheat Carrying *Pm40* Response to *Blumeria graminis* f. sp. *tritici* Using Two-Dimensional Electrophoresis

**DOI:** 10.3390/ijms20040933

**Published:** 2019-02-21

**Authors:** Yinping Liang, Ye Xia, Xiaoli Chang, Guoshu Gong, Jizhi Yang, Yuting Hu, Madison Cahill, Liya Luo, Tao Li, Lu He, Min Zhang

**Affiliations:** 1 College of Agronomy & Key Laboratory for Major Crop Diseases, Sichuan Agricultural University, Chengdu 611130, China; liangyinping3@163.com (Y.L.); xl_changkit@126.com (X.C.); guoshugong@126.com (G.G.); yjzgxy@126.com (J.Y.); yutinghu116sicau@163.com (Y.H.); scmlsjc@163.com (L.L.); 13187874590@126.com (T.L.); hl415676745@163.com (L.H.); 2 Department of Plant Pathology, The Ohio State University, Columbus, OH 43202, USA; xia.374@osu.edu (Y.X.); madisontaylor313@hotmail.com (M.C.); 3 Institute of Ecological Agriculture, Sichuan Agricultural University, Chengdu 611130, China

**Keywords:** *Blumeria graminis* f. sp. *tritici*, protein two-dimensional electrophoresis, mass spectrometry, *Pm40*

## Abstract

Wheat powdery mildew caused by *Blumeria graminis* f. sp. *tritici* (*Bgt*) is considered a major wheat leaf disease in the main wheat producing regions of the world. Although many resistant wheat cultivars to this disease have been developed, little is known about their resistance mechanisms. *Pm40* is a broad, effective resistance gene against powdery mildew in wheat line *L699*. The aim of this study was to investigate the resistance proteins after *Bgt* inoculation in wheat lines *L699*, *Neimai836*, and *Chuannong26*. *Neimai836* with *Pm21* was used as the resistant control, and *Chuannong26* without any effective *Pm* genes was the susceptible control. Proteins were extracted from wheat leaves sampled 2, 4, 8, 12, and 24 h after *Bgt* inoculation, separated by two-dimensional electrophoresis, and stained with Coomassie brilliant blue G-250. The results showed that different proteins were upregulated and downregulated in three wheat cultivars at different time points. For the wheat cultivar *L699*, a total of 62 proteins were upregulated and 71 proteins were downregulated after *Bgt* inoculation. Among these, 46 upregulated proteins were identified by mass spectrometry analysis using the NCBI nr database of *Triticum*. The identified proteins were predicted to be associated with the defense response, photosynthesis, signal transduction, carbohydrate metabolism, energy pathway, protein turnover, and cell structure functions. It is inferred that the proteins are not only involved in defense response, but also other physiological and cellular processes to confer wheat resistance against *Bgt*. Therefore, the resistance products potentially mediate the immune response and coordinate other physiological and cellular processes during the resistance response to *Bgt*. The lipoxygenase, glucan exohydrolase, glucose adenylyltransferasesmall, phosphoribulokinase, and phosphoglucomutase are first reported to be involved in the interactions of wheat-*Bgt* at early stage. The further study of these proteins will deepen our understanding of their detailed functions and potentially develop more efficient disease control strategies.

## 1. Introduction

Wheat powdery mildew caused by the obligate fungus *Blumeria graminis* f. sp. *tritici (Bgt)* is a major wheat leaf disease in the main wheat producing regions of world, leading to significant yield loss each year [1]. Agricultural and chemical methods are widely used to combat the disease. Wheat powdery mildew is an airborne disease and the chemical control methods for *Bgt* seriously pollute environments. Therefore, planting resistant cultivars is the most economical, most effective, and safest method to control wheat powdery mildew [2]. To date, approximately 90 formally designated powdery mildew resistance genes (*Pm* genes) are catalogued at 58 loci (*Pm1*–*Pm62*, *Pm18* = *Pm1c*, *Pm22* = *Pm1e*, *Pm23* = *Pm4c*, *Pm31* = *Pm21*) with the loci of *Pm1*, *Pm3*, *Pm4*, *Pm5*, and *Pm24* having 5, 17, 4, 5, and 2 alleles, respectively [3,4,5,6,7,8,9,10,11,12,13]. However, resistance genes often become ineffective due to the enrichment and variation of virulent races, particularly when a single resistance gene is used in large areas for long periods of time. Therefore, it is very important to identify effective resistance genes and develop multiple resistance cultivars in wheat breeding [14].

The resistant mechanisms of wheat cultivars against *Bgt* are not well-known. Bread wheat (*Triticum aestivum* L.) is a hexaploid (2*n* = 42; AABBDD) with a 17-gigabase genome that contains 124 201 genes [15]. Due to this complexity, cloning wheat genes by the standard map-based cloning strategy remains challenging. Although many powdery mildew resistance genes were identified and mapped in wheat, to date, only five genes, *Pm2*, *Pm3*, *Pm8*, *Pm21*, and *Pm60* have been cloned [9,16,17,18,19,20]. The resistance gene *Pm40* was transferred from *Elytrigia intermedium* into wheat line *GRY19* and mapped on chromosome arm 7BS [21]. *Pm40* is flanked by *Xwmc335* and *BF291338* at genetic distances of 0.58 cM and 0.26 cM, respectively, in deletion bin C-7BS-1-0.27 [22]. Wheat line *L699*, which is the high generation of wheat line *GRY19*, carries the resistance gene *Pm40* and confers resistance to all available isolates of *Bgt* in China [23].

Proteins are not only the final executant of life functions but also the key to understanding physiological, pathological, and pharmacological functions of plants [24]. Therefore, it is difficult to thoroughly explain the powdery mildew resistance mechanism using genomic and transcriptomic methods. Proteomic approaches have been extensively applied in plant pathology research [25,26]. However, only a few studies examined the changes of plant proteome in response to *Bgt*. Wheat cultivars Bainong/W2132 (*Pm21*), JD8/JD8-*Pm30*, N8038 (*PmG25*), N9134 (*PmAS846*), and Xinong979 (without effective *Pm* genes) were used to analyze the effect of *Bgt* on wheat protein expression. These studies showed that most of the upregulated proteins were involved in stress responses and primary metabolic pathways [24,27,28,29,30]. However, there is no such study investigating the differences of protein expressions in the period before *Bgt* haustoria formation, which is very critical for us to better understand the interactions of this pathogen with different wheat cultivars at early stage. To understand the molecular recognition of wheat-*Bgt* during the contact period and penetration period, we identified a set of proteins in wheat inoculated with *Bgt* using two-dimensional electrophoresis (2-DE). The possible roles of the identified proteins in the defense response at early interaction stage were discussed according to their functional implications. This study deepens and extends our knowledge on the interactions of wheat with *Bgt* and allows us to further understand the wheat immune systems against *Bgt*. All these will facilitate the development of more efficient strategies to control this devastating pathogen for enhancing wheat production, which can also potentially provide insights for the control of different plant diseases caused by diverse powdery mildew pathogens.

## 2. Results

### 2.1. Phenotypic Differences of Leaves Affected by Bgt

**Figure 1:**
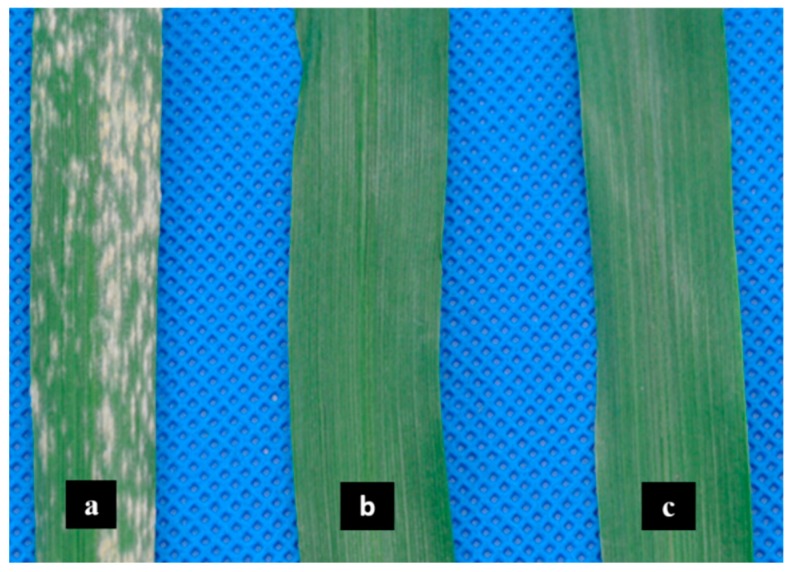
Different responses of wheat leaves to *Blumeria graminis* f. sp. *tritici (Bgt)* infection after six days. (**a**) Susceptible wheat cultivar *Chuannong26*. (**b**) Resistant wheat cultivar *L699*. (**c**) Resistant wheat cultivar *Neimai836*.

The bioassay revealed differences in resistance to *Bgt* among *L699*, *Chuannong26* and *Neimai836* (Figure 1). The susceptible cultivar *Chuannong26* was covered by a high number of sori and had the white powdery appearance due to the abundant conidia and conidiophores production on the leaf surface after 6 days of *Bgt* infection, with the infection type (IT) = 9 (Figure 1a). Meanwhile, the resistant wheat lines *L699* and *Neimai836* were observed to be healthy without any epidermal cell necrosis, chlorotic patches, and powdery appearance on the leaf surface, with the IT = 0 (*L699*: Figure 1b, *Neimai836*: Figure 1c) [23].

### 2.2. Estimation of Wheat-Bgt Interactions

**Figure 2:**
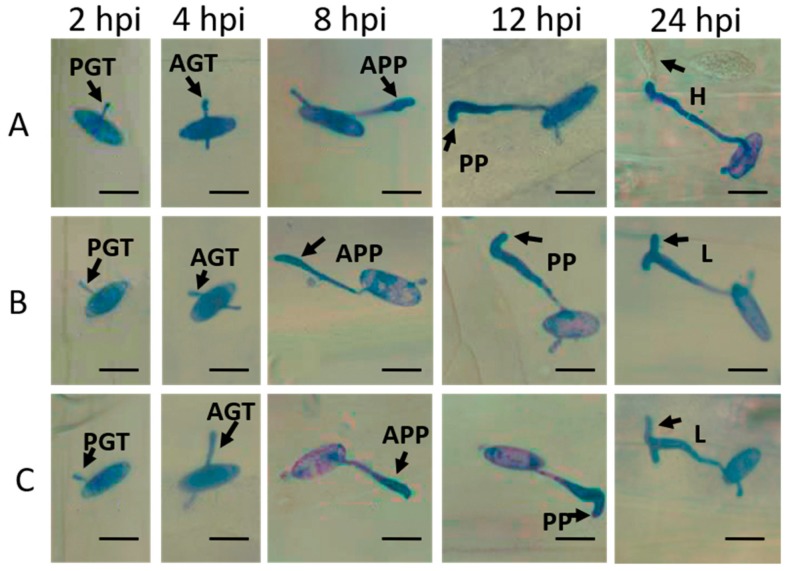
Microscopic observations of wheat-*Bgt* interactions on the leaf surface. The development of *Bgt* at 2, 4, 8, 12, and 24 hpi in wheat cultivars *Chuannong26* (**A**), *L699* (**B**), and *Neimai836* (**C**). PGT: primary germ tube, AGT: appressorium germ tube, APP: appressorium, PP: penetration peg, H: haustorium and L: lobe. Scale bar: 20 μm.

To examine the development of *Bgt* and immune responses of wheat at 2, 4, 8, 12, and 24 h post-inoculation (hpi), the cytological observations of wheat samples were carried out. *Bgt* conidia successively formed primary germ tubes, appressorium germ tubes, appressoria, penetration pegs, and haustoria at 2, 4, 8, 12, and 24 hpi in susceptible wheat cultivar *Chuannong26*. However, in resistant wheat cultivars *L699* and *Neimai836*, only a small number of conidia successfully penetrated the epidermal cells at 24 hpi, and the hypersensitive reaction (HR) and formation of papilla (PA) effectively suppressed the development of haustoria and hyphae [31]. In addition, the appressoria of some conidia sprouted another lobe and stopped growing because of the lack of nutrition at 24 hpi (Figure 2).

### 2.3. Detection of Differential Proteins by 2-DE

**Figure 3:**
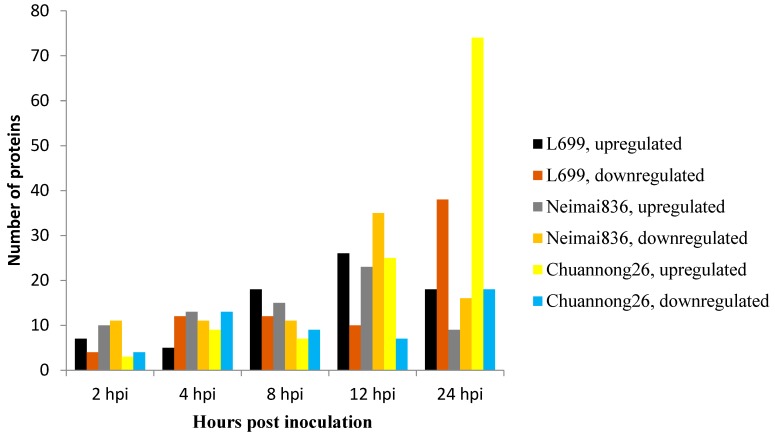
Number of proteins differentially expressed after powdery mildew infection at different time points.

**Figure 4:**
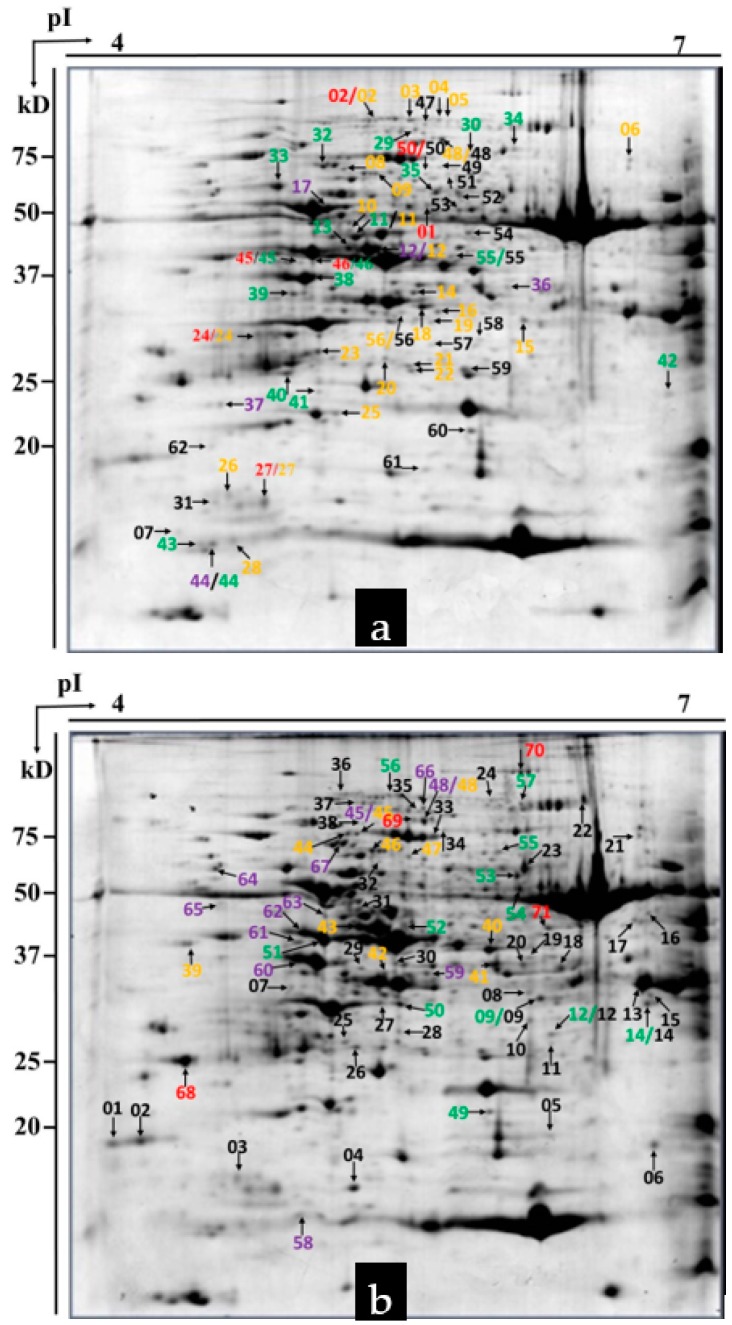
Differences in protein expression between resistant wheat cultivar *L699* wheat with and without powdery mildew infection. (**a**) Upregulated proteins are labeled in the representative 2-DE gel of *Bgt*-inoculated *L699* wheat at 24 hpi. (**b**) Downregulated proteins are labeled in the representative 2-DE gel of mock-inoculated *L699* wheat at 24 hpi. Red, 2 hpi; purple, 4 hpi; green, 8 hpi; yellow, 12 hpi; black, 24 hpi.

**Figure 5:**
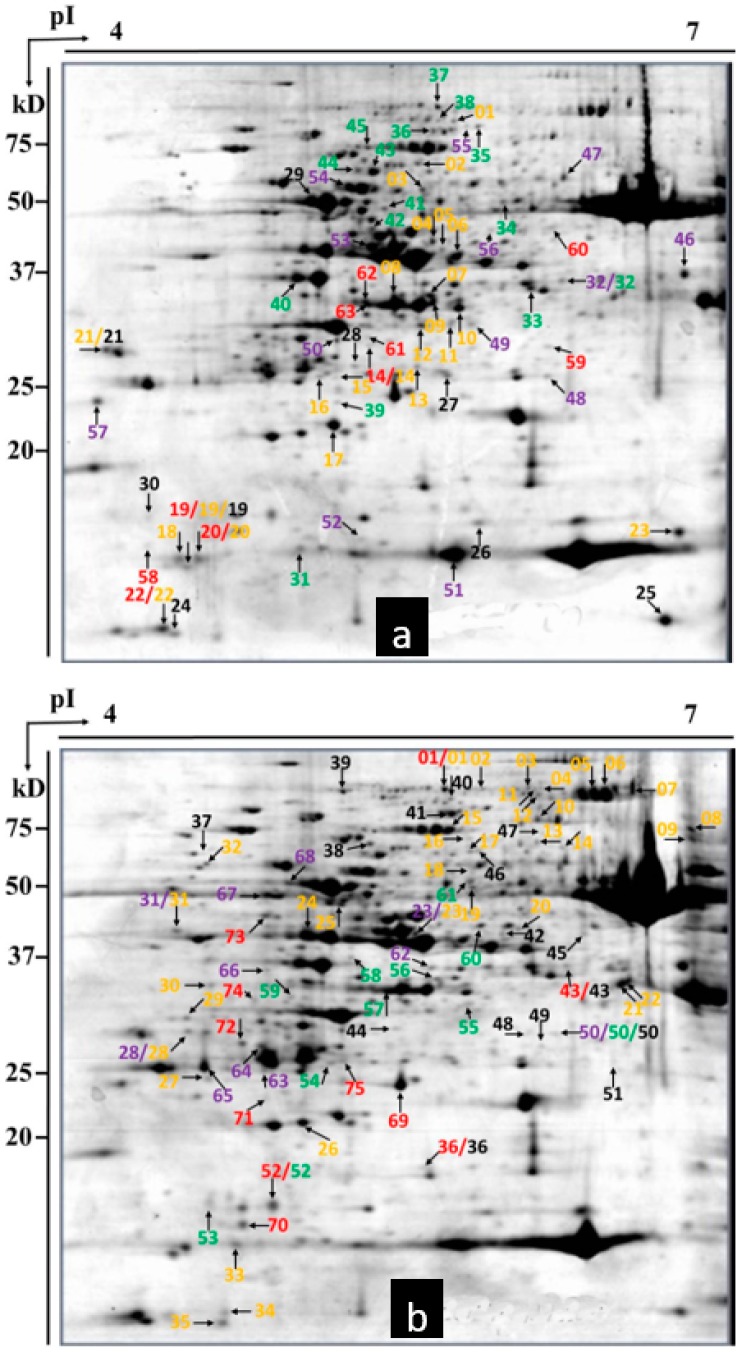
Differences in protein expression between resistant wheat cultivar *Neimai836* wheat with and without powdery mildew infection. (**a**) Upregulated proteins are labeled in the representative 2-DE gel of *Bgt*-inoculated *Neimai836* wheat at 12 hpi. (**b**) Downregulated proteins are labeled in the representative 2-DE gel of mock-inoculated *Neimai836* wheat at 12 hpi. Red, 2 hpi; purple, 4 hpi; green, 8 hpi; yellow, 12 hpi; black, 24 hpi.

**Figure 6:**
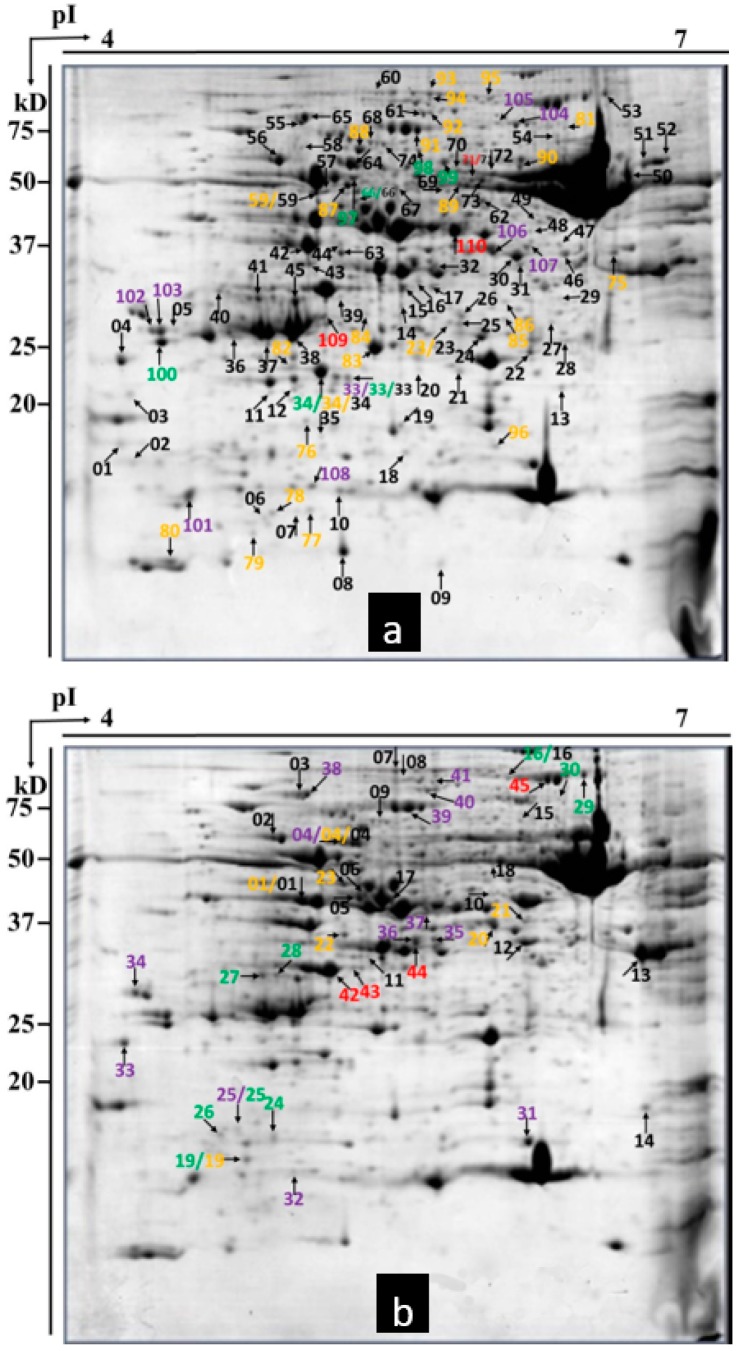
Differences in protein expression between susceptible wheat cultivar *Chuannong26* wheat with and without powdery mildew infection. (**a**) Upregulated proteins are labeled in the representative 2-DE gel of *Bgt*-inoculated *Chuannong26* wheat at 24 hpi. (**b**) Downregulated proteins are labeled in the representative 2-DE gel of mock-inoculated *Chuangnong26* wheat at 24 hpi. Red, 2 hpi; purple, 4 hpi; green, 8 hpi; yellow, 12 hpi; black, 24 hpi.

Approximately 500 protein spots were detected in all gels in this study. Using a twofold change cutoff, we found wheat cultivars *L699*, *Neimai836*, and *Chuannong26* all had upregulated and downregulated proteins affected by *Bgt*. The numbers of upregulated proteins were seven (spot 01, 02, 24, 27, 45, 46, 50), five (spot 12, 17, 36, 37, 44), 18 (spot 11, 13, 29, 30, 32–35, 38–46, 55), 26 (spot 02–06, 08–12, 14–16, 18–28, 48, 56), and 18 (spot 07, 31, 47–62). The numbers of downregulated proteins were four (spot 68–71), 12 (spot 45, 48, 58–67), 12 (spot 09, 12, 14, 49–57), 10 (spot 39–48), and 38 (spot 01–38) in wheat cultivar *L699* at 2, 4, 8, 12, 24 hpi, respectively (Figure 3 and Figure 4). The numbers of upregulated proteins were 10 (spot 14, 19, 20, 22, 58–63), 13 (spot 32, 46–57), 15 (spot 31–45), 23 (spot 1–23), and nine (spot 19, 21, 24–30). The numbers of downregulated proteins were 11 (spot 01, 36, 43, 52, 69–75), 11 (spot 23, 28, 31, 50, 62–68), 11 (spot 50, 52–61), 35 (spot 1–35), and 16 (spot 36–51) in wheat cultivar *Neimai836* at 2, 4, 8, 12, 24 hpi, respectively (Figure 3 and Figure 5). The numbers of upregulated proteins were three (spot 71, 109–110), nine (spot 33, 101–108), seven (spot 33, 34, 66, 97–100), 25 (spot 23, 34, 59, 75–96), and 74 (spot 1–74). The numbers of downregulated proteins were four (spot 42–45), 13 (spot 04, 25, 31–41), nine (spot 16, 19, 24–30), 7 (spot 01, 04, 19–23), and 18 (spot 1–18) in wheat cultivar *Chuannong26* at 2, 4, 8, 12, 24 hpi, respectively (Figure 3 and Figure 6).

### 2.4. Protein Identification

Sixty-two upregulated proteins in *L699* at five different inoculation time points were eluted from representative 2-DE gels for identification, and 46 were successfully identified. Bioinformatics analysis of the identified proteins revealed that these proteins were putatively involved in diverse biological processes including stress and disease resistance, photosynthesis, signal transduction, carbohydrate metabolism, energy pathway, gene expression, protein turnover, and cell structure (Table 1). Five proteins (approximately 16.5% of the total differentially expressed proteins (DEPs)) were unnamed or hypothetical proteins. The largest category of these upregulated proteins was protein turnover (28%, thirteen), followed by carbohydrate metabolism (22%, ten), stress and disease resistance (13%, six), photosynthesis (13%, six), energy pathway (6.5%, three), signal transduction (2%, one), gene expression (2%, one), and cell structure (2%, one).

### 2.5. Validation of Upregulated Proteins by qRT-PCR

**Figure 7:**
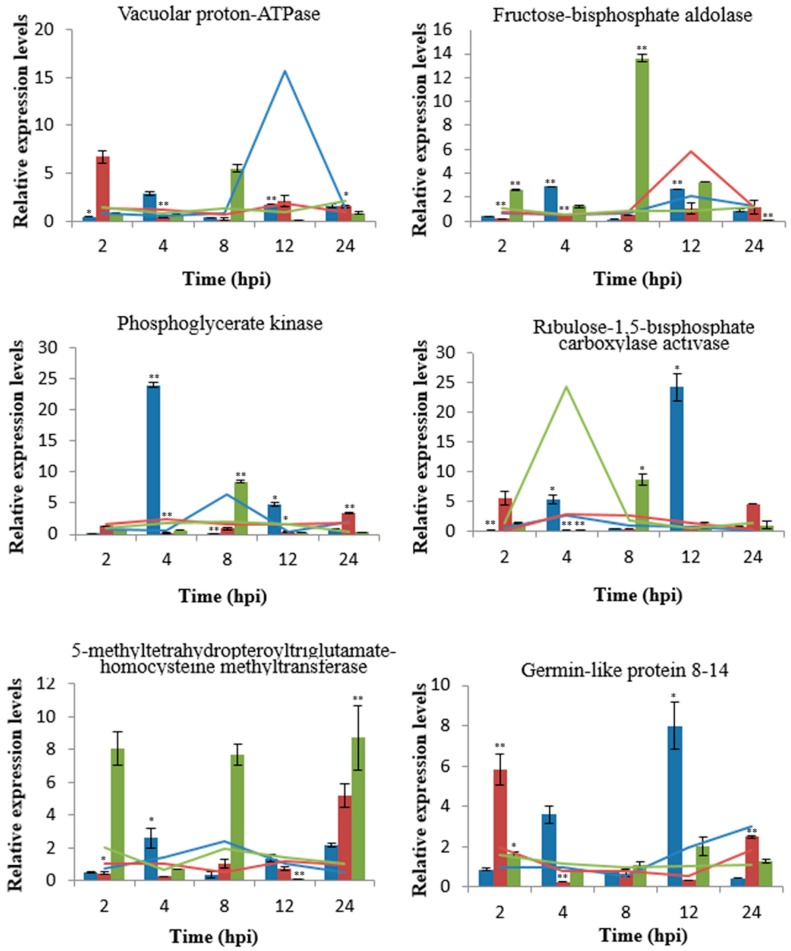
Quantification of six gene transcripts and protein levels at different time points post inoculation with *Bgt* in wheat. The bar graph shows the fold changes of the mRNA expression levels in inoculation vs control samples at five time points. The blue, red, and green columns are representatives of wheat *L699*, *Neimai836*, and *Chuannong26*, respectively. The lines show the fold changes of the protein expression levels in inoculation vs control samples at five time points. The blue, red, and green lines are representatives of wheat *L699*, *Neimai836*, and *Chuannong26*, respectively. The mRNA expression levels were quantified by qRT-PCR normalized against *18S rRNA*. Asterisks indicate statistically significant differences (*, *p* < 0.05; **, *p* < 0.01) of mRNA expression levels between the inoculation and control samples.

To confirm the changes in protein abundance, qRT-PCR was used to analyze the mRNA expression levels of protein-coding genes after inoculation with *Bgt* in wheat cultivars *L699*, *Neimai836*, and *Chuannong26*. Six upregulated proteins were randomly selected and primers for the mRNAs of these proteins were specifically designed (shown in Table 2). The mRNA levels for all the six proteins were significantly increased in at least one sampling time in wheat *L699*. These changes reflected the increases in proteins and the differentially expressed proteins identified by two-dimensional electrophoresis were validated (Figure 7). However, the mRNAs of four proteins, i.e. fructose-bisphosphate aldolase, phosphoglycerate kinase, 5-methyltetrahydropteroyltriglutamate-homocysteine methyltransferase, and the Germin-like protein (GLP) 8-14, were upregulated earlier than proteins in wheat *L699*. The protein level of fructose-bisphosphate aldolase increased at 12 hpi, but the mRNA level did not exhibit a significant increase at any sampling time point in wheat *Neimai836*.

## 3. Discussion

Plants employ two levels of immunity to encounter pathogen invasion: Pathogen-associated molecular pattern (PAMP)-triggered immunity (PTI) and effector-triggered immunity (ETI). In the early phase of defense, PAPMs are recognized as ‘non-self’ molecules by the host plants. This induces downstream defense signaling, such as the generation of reactive oxygen species (ROS) and the transcription of genes encoding pathogenesis-related proteins (PRs). The pathogens release effector proteins to oppose PTI, and then the plant resistance proteins recognize the effector, which stimulates the plant’s ETI, leading to the hypersensitive response (HR) and activating other plant defense pathways [32,33,34].

However, not only the specific signaling mediated by resistance genes, but also the other basic cellular processes, are involved in the effective defense to support the plant innate immune system [35]. In our results, the differentially expressed proteins, including both resistance proteins against *Bgt* and other proteins related to the direct and indirect defensive processes. The potential roles of these proteins in the defense response are discussed below.

### 3.1. Stress- and Defense-Related Proteins

Plants experience a variety of biotic and abiotic stresses during the growth and development periods. Studies on the plant stress response found many stress response related proteins. For example, the Germin-like proteins are important stress-related proteins.

Protein spot 61, with an increasing expression level 24 h after *Bgt* infection, was identified as GLPs. GLPs as extracellular glycoproteins are important components of the plant PRs [36]. Recently, GLPs were reported to be involved in the stress responses of Arabidopsis, pepper, barely, and rice [37,38,39,40]. GLPs can remove excess ROS generated by plants in the form of enzymes, receptors, or structural proteins in various physiological and biochemical processes. The expression of GLPs increased significantly and potentially catalyzing the production of H_2_O_2_, in plants infected by fungi, bacteria, viruses, or other pathogens [41,42,43]. H_2_O_2_ can selectively participate in the signaling cascade pathway, which can stimulate plant self-defense reactions. In addition, H_2_O_2_ is able to use the cellulose crosslinking action to strengthen the structure of plant cell walls, which is very important in plant defense against oxidative stress. GLPs play an important role in wheat *L699* resistance to powdery mildew. This result is consistent with the previous study [43].

Heat shock proteins, as chaperones during the stress response, are very important for the correct folding of newly synthesized proteins [44]. Heat shock proteins were first discovered in Drosophila and were a class of proteins expressed by organisms under high temperature stimulation [45,46]. Recently, heat shock proteins were found to have very important roles in the innate immune response and are indispensable for the function of other defense-related proteins [47,48]. Mandal found that heat shock protein expression was significantly increased in wheat *N0308* 72 h after *Bgt* infection [29]. Protein spot 32 was identified as heat shock proteins with an expression level that increased after 8 h with *Bgt* inoculation. Heat shock proteins are closely related to wheat resistance of powdery mildew, as reported previously [29].

Plant lipoxygenases are members of a class of nonheme iron-containing dioxygenases that catalyze the addition of molecular oxygen to fatty acids containing a cis, cis-l,4-pentadiene system, which produces an unsaturated fatty acid hydroperoxide [49]. Currently, an increasing number of studies show that there are many similarities between the plant defense mechanisms and the animal defense mechanisms under adverse conditions [50]. Lipoxygenases in animals and plants play an important role in withstanding adverse environments. Protein spots 3, 4, 5, and 29, which were identified as lipoxygenase, were upregulated 8 h and 12 h after *Bgt* inoculation compared to non-inoculated wheat. In the lipoxygenase pathway, the polyunsaturated fatty acids are catalyzed by lipoxygenases to generate hydrogen peroxide and subsequently form compounds with a specific mass of physiological functions by the catalytic reaction of other enzymes, such as jasmonic acid and guaiac acid, which induces the synthesis of resistance proteins against stresses [51,52].

### 3.2. Proteins Related to Photosynthesis

Plant defense reactions are closely related to photosynthesis. It is generally believed that plant photosynthesis-related protein biosynthesis is reduced and resources are allocated to the defense response when plants are infected by a pathogen. The plant defense responses to pathogens is known as the “hidden costs” defense [53]. Plants affected by pathogens active the HR response, which is considered as another reason for weakening the plant photosynthesis after the original infection. However, protein spots 11, 28, 33, 36, 44 comprise ribulose carboxylase, which is an indicator of photosynthesis. These spots were upregulated 4 h, 8 h, and 12 h after powdery mildew infection, which could indicate that photosynthesis is increased. It was reported that photosynthesis is enhanced in early plant pathogen infections and weakened on later stage during the infection [54].

### 3.3. Proteins Involved in Carbohydrate Metabolism and Energy Pathways

The expression of several proteins involved in glucose metabolism, including β-d-glucose hydrolase (spot 6), phosphoglycerate kinase (spot 13, 55), glycerol phosphodiester enzyme (spot 14), diphosphate aldolase (spot 18), ribulose kinase (spot 38), glucose phosphate mutase (50, 51), and six-glucose phosphate decarboxylase (spot 54), were increased in response to wheat powdery mildew in wheat *L699*. Previous studies have shown that hexose can provide extra energy and serve as a signal for activating resistant response. For instance, in response to barley powdery mildew infection, the expression of hexose metabolizing enzymes significantly increased [55].

### 3.4. Proteins Involved in Gene Expression and Protein Turnover

Each step in the flow of genetic information is very strict, so the error rate of protein synthesis in this process is very low. However, protein synthesis has a certain error rate that is the net result of several processes. Aminoacyl-tRNA synthetases and ribosomes play important roles in protein synthesis [56,57]. Studies have shown that aminoacy-tRNA synthetase is not only involved in protein synthesis, but also participates in other activities, including the regulation of transcription and translation, RNA splicing, signal transduction, and immune response [58]. Current research is focused on the relationship between the function and structure of new amino acid-tRNA synthetases, especially the aminoacyl-tRNA synthetase, which is related to diseases. After powdery mildew infection in wheat *L699*, the expression levels of alanyl-tRNA synthetase (spot47), lysyl-tRNA synthetase (spot49), and ribosomal protein (spot27) increased, suggesting that these proteins may be important in the wheat anti-powdery mildew responses.

### 3.5. Proteins Associated with Cell Organization

In powdery mildew-infected wheat *L699*, actin (spot 12) was upregulated. The actin cytoskeleton is an essential dynamic component for cells and is highly conserved in eukaryotic cells. The cytoskeleton is closely linked with the membrane and is involved in various cellular processes, including defense signaling based on actin cytoskeletal structures after pathogen infection [59].

### 3.6. The Correlation of mRNA and Protein Expression

The analysis of six proteins and the expression of the protein-coding regions of their genes showed that the proteins and mRNA levels had a certain uniformity in our study. However, there exists post-transcriptional regulation after translational regulation in wheat, which may lead to differences between protein expression levels and mRNA levels.

### 3.7. The Novel Proteins Potentially Involed in the Response of Wheat Against Bgt

Some of the identified proteins, such as the lipoxygenase, glucan exohydrolase, glucose adenylyltransferasesmall, phosphoribulokinase, and phosphoglucomutase, are first reported during the interaction of wheat-*Bgt* in this study. These proteins are potentially very critical for the wheat-*Bgt* interaction at early stage. For future research, the defense functions of these novel proteins deserve further investigation by using the integrative approaches, such as the comparative metabolomics, gene overexpression, and silencing methods. The related study will lead to deeper understanding of the detailed functions of these important proteins and more efficient disease control strategies.

## 4. Materials and Methods

### 4.1. Plant Materials and Inoculation

*L699*, *Neimai836*, and *Chuannong26* were used in this study. *L699* carries the resistance gene *Pm40* and shows resistance to most powdery mildew isolates in China. *Neimai836* carries the resistance gene *Pm21*, but not *Pm40*, and also shows resistance to most powdery mildew isolates in China. Nevertheless, *Chuannong26* is highly susceptible to powdery mildew without any effective resistance gene. Plants were cultivated in 30 cm pots in a growth chamber at 18 °C under a 12 h/12 h dark photoperiod. These pots were divided into the *Bgt*-inoculated group and the mock-inoculated group with nonopaque and breathable hoods. Seedlings of the *Bgt*-inoculated group were artificially inoculated by dusting with *Bgt* conidia from sporulating seedlings of *Chuannong26* at two to three leaf stages. Leaf samples were harvested at 2, 4, 8, 12, and 24 hpi with liquid nitrogen and immediately stored at −80 °C. Samples were collected from three biological replicates at each time point and every sample protein was run on three gels. For the analysis, one best gel was selected from three gels.

### 4.2. Cytological Observation of the Interaction between Wheat and Bgt

Leaves of the *Bgt*-inoculated and mock-inoculated wheat were sampled at 2, 4, 8, 12, and 24 hpi and cut into 2–3 cm leaf fragments. The leaf fragments collected at 2, 4, 8, and 12 hpi were destained using isopropanol fumigation. The leaf fragments collected at 24 hpi were destained with AA solution (ethanol:glacial acetic acid = 1:1, *v*/*v*). Then the sample were stained with Coomassie blue staining solution (0.15% trichloroacetic acid aqueous solution:0.6% Coomassie brilliant blue R-250 methanol solution = 1:1, *v*/*v*) for 4 h. After rinsing with distilled water, the leaves were saved in a mix solution (glacial acetic acid:glycerol:distilled water = 1:4:15, *v*/*v*/*v*). The infection structures including germ tubes, appressoria and haustoria of *Bgt* were observed under electron microscope (40×, Nikon Eclipse 80i, Nikon Corporation, Tokyo, Japan).

### 4.3. Protein Extraction

Leaf tissue (1 g) was ground in a prechilled mortar with liquid nitrogen. Then, the powder was transferred to a 1.5 mL centrifuge tube with the addition of 1 mL acetone containing 10% trichloroacetic acid (TCA) and 0.07% β-mercaptoethanol. The samples were vortexed and chilled for 1 h at −20 °C. Then the homogenate was centrifuged at 13,000× *g* for 30 min at 4 °C. After the supernatant was gently decanted, the pellet was washed four times with chilled acetone containing 0.07% β-mercaptoethanol, then dried until all the acetone was removed by a vacuum drying instrument. The resulting powder was dissolved in 1 mL of IEF buffer (7 M urea, 2 M thiourea, 4% CHAPS, 20 mM DTT, 0.001% bromophenol blue, and 0.5% ampholyte (pH 3–10)). After centrifugation at 13,000× *g* for 20 min twice, the leaf proteins were obtained from the supernatant, and their concentration was determined using a Bradford dye binding assay [60].

### 4.4. Two-Dimensional Electrophoresis, Protein Visualization, and Image Analysis

The protein mixture was loaded onto an IPG strip (17 cm, pH 4-7, linear gradient (Bio-Rad, California, CA, USA)) by active rehydration at 50 V for 14 h (20 °C) on a Protein Isoelectric Focusing (IEF) Cell (Bio-Rad). The following conditions were used for the IEF: 20 °C, 50 μA/strip, 250 V for 1 h, 1000 V for 1 h, 10,000 V for 5 h, and 10,000 V, with a total of 60,000 vhs. The focused IPG strips were equilibrated in buffer containing 5 mL 6 M urea, 2% SDS, 20% glycerol, 375 mM Tris-HCl (pH 8.8), and 200 mM DTT for 15 min, then re-equilibrated in a similar buffer whose 200 mM DTT was replaced by 250 mM iodoacetamide for 15 min. Proteins were separated on the second dimension on vertical 12% sodium dodecyl sulfate-polyacrylamide (SDS-PAGE) gel in a Protean II XI Cell (Bio-Rad) at 25 mA/gel. Proteins in the gels were stained by the “blue silver” protocol as described by Candiano et al. [61]. Gels were scanned by a GS-800 scanner (Bio-Rad) and the proteins in the images were analyzed using PDQuest software with version 8.0 (Bio-Rad). There were variations due to sample loading, the 2-DE techniques and staining. To minimize these variations, each spot intensity was normalized according to its percent volume of all protein spots on the gel. The proteins showing at least a twofold change in abundance were considered as differentially expressed proteins (DEPs).

### 4.5. MS and Database Searches

Protein slices in fresh blue silver-stained gel were excised and plated into a 96-well microtiter plate. Excised slices were first distained twice with 60 μL 50 mM NH_4_HCO_3_ and 50% acetonitrile, then dried twice with 60 μL acetonitrile. Afterwards, the dried pieces of gels were incubated in ice-cold digestion solution (12.5 ng/μL trypsin and 20 mM NH_4_HCO_3_) for 20 min, then transferred into a 37 °C incubator for digestion overnight. Finally, peptides in the supernatant were collected after extraction twice with 60 μL extraction solution (5% formic acid in 50% acetonitrile).

The peptide solution described above was dried under the protection of N_2_. A 0.8 μL matrix solution (5 mg/mL α-cyano-4-hydroxy-cinnamic acid diluted in 0.1% TFA, 50% ACN) was pipetted to dissolve the peptides. Then, the mixture was spotted on a MALDI target plate (AB SCIEX, Framingham, Massachusetts, MA, USA). MS analysis of the peptides was performed on an AB SCIEX 5800 TOF/TOF. The UV laser was operated at a 400 Hz repetition rate with a wavelength of 355 nm. The accelerated voltage was operated at 20 kV, and the mass resolution was maximized at 1600 Da. The mass instrument with internal calibration mode was calibrated by myoglobin digested with trypsin. All acquired spectra of samples were processed using TOF/TOF Explorer^TM^ Software (AB SCIEX) in default mode. The data were searched by GPS Explorer (V3.6) with the search engine MASCOT (V2.3, Matrix Science, London, UK). The search parameters were as follows: dates were compared against the NCBI nr database, trypsin was digested with one missing cleavage, MS tolerance was set at 100 ppm, and MS/MS tolerance was set to 0.6 Da. Functional annotation of identified proteins based on gene ontology was performed using the Protein Information Resource (https://proteininformationresource.org).

### 4.6. RNA Isolation and qRT-PCR Assays

Total RNA from *Bgt*-inoculated or mock-inoculated wheat leaves was sampled at 2, 4, 8, 12, and 24 hpi and extracted using Trizol reagent (Tiangen Biotech, Beijing, China). First strand cDNA was synthesized with Transcript One-Step gDNA removal and cDNA Synthesis Supermix (Transgen Biotech, Beijing, China). Primers were specifically designed to anneal to each of the selected genes and the endogenous reference gene *18S rRNA* (GenBank accession No. AY049040) [62]. The expression patterns of selected genes were analyzed with a Bio-Rad iQ5 system. Relative gene quantification was calculated by the comparative 2^–ΔΔ*C*t^ method [63] and normalized to the corresponding expression level of the *18S rRNA*. All reactions were performed in triplicate, including three no-template controls.

## 5. Conclusions

In summary, we identified 46 differentially expressed proteins in wheat in response to *Bgt* inoculation using 2-DE and mass spectrometry. Among these identified proteins, the lipoxygenase, glucan exohydrolase, glucose adenylyltransferasesmall, phosphoribulokinase, and phosphoglucomutase are first reported during the interaction of wheat-*Bgt*. We inferred that these proteins are not only involved in defense response but also physiology and cellular process for wheat to confer resistance against *Bgt*. The wheat resistance gene products potentially mediate the immune response and coordinate other physiological and cellular processes during the resistance response to *Bgt*.

## Figures and Tables

**Table 1:** Identification of differentially upregulated proteins in wheat resistance cultivar *L699* by MALDI-TOF-MS.

Spot	Protein Name	Accession	Matched Peptides	MW/PI	Score	Time (h)
	Proteins involved in disease defense response					
3	Lipoxygenase 2.1, chloroplastic	gi|473948122	21	105625.33/5.70	185	12
4	Lipoxygenase 2.1, chloroplastic	gi|473948122	41	105625.33/5.70	591	12
5	Lipoxygenase 2.1, chloroplastic	gi|473948122	17	105625.33/5.70	196	12
29	Lipoxygenase 1	gi|474399175	16	96333.65/5.91	299	8
32	Heat shock cognate 70 kDa protein 1	gi|474012573	37	71123.58/5.06	641	8
61	Germin-like protein 8-14	gi|473963025	4	21939.25/5.36	174	24
	Photosynthesis-related proteins					
11	Ribulose-1,5-bisphosphate carboxylase activase, partial	gi|37783283	10	22336.08/4.98	309	8, 12
28	Ribulose bisphosphate carboxylase small chain, chloroplastic	gi|473882355	14	18526.35/8.65	208	12
33	RuBisCO large subunit-binding protein subunit alpha, chloroplastic	gi|474113969	34	65380.60/5.17	864	8
36	Ribulose-1,5-bisphosphate carboxylase activase, partial	gi|37783283	10	22336.08/4.98	330	4
37	Photosystem II cytochrome b559 alpha subunit (chloroplast)	gi|699976019	6	9444.60/4.64	196	4
44	Ribulose bisphosphate carboxylase small chain PWS4.3, chloroplastic	gi|132087	2	19417.36/8.99	92	4, 8
	Proteins involved in Signal transduction					
24	14-3-3 protein	gi|431822520	16	29264.88/4.83	434	12
	Carbohydrate metabolism-related proteins					
6	Beta-d-glucan exohydrolase	gi|20259685	14	67301.15/6.87	74	12
10	Glucose-1-phosphate adenylyltransferasesmall subunit, chloroplastic/amyloplastic	gi|474108293	23	64723.14/7.91	266	12
13	Phosphoglycerate kinase	gi|3293043	16	49839.53/6.57	580	8, 12
14	Glycerophosphodiester phosphodiesterase GDE1	gi|473847956	13	52899.68/5.69	43	12
18	Fructose-bisphosphate aldolase, chloroplastic	gi|473848356	15	42002.99/5.94	358	12
38	Phosphoribulokinase	gi|5924030	22	45141.39/5.72	587	8
50	Phosphoglucomutase, cytoplasmic	gi|473763033	18	63499.68/5.14	302	2, 24
51	Phosphoglucomutase, partial	gi|18076790	15	62789.15/5.66	218	24
54	6-phosphogluconate dehydrogenase, decarboxylating	gi|474379872	23	81169.95/8.56	608	24
55	Cytosolic 3-phosphoglycerate kinase, partial	gi|28172911	16	31334.35/4.98	291	8, 24
	Proteins involved in energy pathway					
9	Vacuolar proton-ATPase subunit A	gi|90025017	37	68454.90/5.23	583	12
17	ATP synthase CF1 beta subunit (chloroplast)	gi|667669997	33	53857.48/5.06	1200	4
39	Ferredoxin-NADP(H) oxidoreductase	gi|20302473	10	40232.03/6.92	120	8
	Proteins involved in gene expression and DNA remodeling					
15	Guanine nucleotide-binding protein subunit beta-like	gi|473957859	6	27150.69/6.29	211	12
	Proteins involved in protein turnover					
8	ATP-dependent zinc metalloprotease FTSH 1, chloroplastic	gi|474350516	29	54477.49/5.58	673	12
27	50S Ribosomal protein L12-2, chloroplastic	gi|475532245	10	21837.90/5.35	452	12
30	Tyrosine phosphorylation protein A	gi|548319365	25	74252.07/6.61	434	8
34	5-methyltetrahydropteroyltriglutamate-Homocysteine methyltransferase	gi|473993302	14	84552.49/5.74	423	8
35	5, 10-methylene-tetrahydrofolate reductase	gi|115589742	12	64875.07/5.86	83	8
40	20 kDa chaperonin, chloroplastic	gi|474407512	10	29710.03/6.76	154	8
47	Putative alanyl-tRNA synthetase, chloroplastic	gi|474142555	12	111648.20/5.62	198	24
48	ATP-dependent Clp protease ATP-binding subunit ClpA-like protein CD4B, chloroplastic	gi|474241774	33	82735.21/5.16	513	12, 24
49	Lysyl-tRNA synthetase	gi|474147702	8	132545.46/6.28	92	24
52	Putative mitochondrial-processing peptidase subunit beta	gi|474142281	30	43290.34/5.41	486	24
53	Adenosylhomocysteinase	gi|474154141	8	45700.84/6.48	56	24
56	Cysteine synthase	gi|474315986	13	35583.27/5.82	216	12, 24
60	Ribosome-recycling factor, chloroplastic	gi|474043078	15	24770.60/8.92	504	24
	Cell structure-related proteins					
12	Actin-3	gi|474259583	18	44367.62/5.26	376	8, 12
	Proteins of unknow function					
2	Hypothetical protein TRIUR3_05354	gi|473755342	27	104676.25/5.87	368	2, 12
26	Unnamed protein product	gi|669029445	4	18152.74/5.60	244	12
58	Hypothetical protein TRIUR3_21449	gi|474384687	14	32942.26/9.31	129	24
59	Unnamed protein product	gi|669027704	13	26818.76/5.57	335	24
63	Unnamed protein product	gi|669029445	5	18152.74/5.60	269	24

**Table 2:** List of primers used for qRT-PCR amplification.

Spot	Protein Name	Accession No.	Primer Sequence 5′-3′
Reference gene	*18S rRNA*	AY049040	Sense: 5′-GTGACGGGTGACGGAGAATT-3′
			Antisense: 5′-GACACTAATGCGCCCGGTAT-3′
9	Vacuolar proton-ATPase	ABD85016	Sense: 5′-TATGAACGTGCTGGGAAGGT-3′
			Antisense: 5′-GGGTTGCAGAGGTAACAGGA-3′
18	Fructose-bisphosphate aldolase	EMS47455	Sense: 5′-TCTTGTCTGGTGGTCAGTCG-3′
			Antisense: 5′-CGTCTTGAGGCAGGTGTTCT-3′
13	Phosphoglycerate kinase	CAA51931	Sense: 5′-AATGGTGCTGTTTTGCTCCT-3′
			Antisense: 5′-TGTTCCGAATGCATCGTTTA-3′
36	Ribulose-1,5-bisphosphate carboxylase activase	AAP72270	Sense: 5′-ACGGACCAGTGACCTTTGAG-3′
			Antisense: 5′-ACCAGTCTTCATCGCATCCT-3′
34	5-methyltetrahydropteroyltriglutamate-homocysteine methyltransferase	EMS51950	Sense: 5′-TGTGTTCTGGTCCAAGATGG-3′
			Antisense: 5′-CTCAAACCTCGGTTGGTCAT-3′
61	Germin-like protein 8-14	EMS51159	Sense: 5′-TGCAGATCACCGACTACGC-3′
			Antisense: 5′-CACGGACTTGAGCTTCTTGAC-3′
